# Supplementary material for: Changes in tooth size of Otaria byronia: an indicator of density-dependent effects?
Source: PeerJ. 2025 Mar 7;13:e18963. doi: 10.7717/peerj.18963 (PMC11892458; doi:10.7717/peerj.18963)
Supplement: Supplemental Information 1 [file peerj-13-18963-s001.docx]

**Supplemental material**

Table S1: Individual Id, Time period and age of South American sea lions used for the analyses.

| **Id** |  | **Time period** | **Age** |
| --- | --- | --- | --- |
| CNP-MAM100075 |  | Postharvest | 14 |
| CNP-MAM100113 |  | Postharvest | 7 |
| CNP-MAM100118 |  | Postharvest | 9 |
| CNP-MAM100122 |  | Postharvest | 12 |
| CNP-MAM100210 |  | Postharvest | 11 |
| CNP-MAM100230 |  | Postharvest | 9 |
| CNP-MAM100251 |  | Postharvest | 8 |
| CNP-MAM100277 |  | Postharvest | 13 |
| CNP-MAM100279 |  | Postharvest | 11 |
| CNP-MAM100315 |  | Postharvest | 11 |
| CNP-MAM100326 |  | Postharvest | 7 |
| CNP-MAM100337 |  | Postharvest | 5 |
| CNP-MAM100350 |  | Postharvest | 12 |
| CNP-MAM100380 |  | Postharvest | 9 |
| CNP-MAM100384 |  | Postharvest | 12 |
| CNP-MAM100399 |  | Postharvest | 7 |
| CNP-MAM100404 |  | Postharvest | 13 |
| CNP-MAM100440 |  | Postharvest | 9 |
| CNP-MAM100458 |  | Postharvest | 8 |
| CNP-MAM100486 |  | Postharvest | 6 |
| CNP-MAM100828 |  | Postharvest | 4 |
| CNP-MAM100841 |  | Postharvest | 6 |
| CNP-MAM100930 |  | Postharvest | 11 |
| CNP-MAM100931 |  | Postharvest | 8 |
| CNP-MAM100986 |  | Postharvest | 12 |
| CNP-MAM100989 |  | Postharvest | 11 |
| CNP-MAM100995 |  | Postharvest | 9 |
| CNP-MAM100997 |  | Postharvest | 9 |
| CNP-MAM101044 |  | Postharvest | 14 |
| CNP-MAM101048 |  | Postharvest | 14 |
| CNP-MAM101061 |  | Postharvest | 8 |
| CNP-MAM101065 |  | Postharvest | 16 |
| CNP-MAM101066 |  | Postharvest | 9 |
| CNP-MAM101088 |  | Postharvest | 11 |
| CNP-MAM101095 |  | Postharvest | 14 |
| CNP-MAM101132 |  | Postharvest | 9 |
| CNP-MAM101139 |  | Postharvest | 7 |
| CNP-MAM101168 |  | Postharvest | 6 |
| CNP-MAM101170 |  | Postharvest | 9 |
| CNP-MAM101180 |  | Postharvest | 7 |
| CNP-MAM101183 |  | Postharvest | 9 |
| CNP-MAM101188 |  | Postharvest | 5 |
| CNP-MAM101192 |  | Postharvest | 13 |
| CNP-MAM101207 |  | Postharvest | 11 |
| CNP-MAM101208 |  | Postharvest | 4 |
| CNP-MAM101279 |  | Postharvest | 7 |
| CNP-MAM101305 |  | Postharvest | 9 |
| CNP-MAM101428 |  | Postharvest | 13 |
| CNP-MAM101431 |  | Harvest | 13 |
| CNP-MAM101432 |  | Harvest | 11 |
| CNP-MAM101438 |  | Postharvest | 11 |
| CNP-MAM101443 |  | Harvest | 7 |
| CNP-MAM101447 |  | Harvest | 4 |
| CNP-MAM101453 |  | Harvest | 8 |
| CNP-MAM101456 |  | Harvest | 5 |
| CNP-MAM101463 |  | Harvest | 11 |
| CNP-MAM101470 |  | Harvest | 10 |
| CNP-MAM101472 |  | Harvest | 7 |
| CNP-MAM101474 |  | Harvest | 7 |
| CNP-MAM101475 |  | Harvest | 7 |
| CNP-MAM101480 |  | Harvest | 11 |
| CNP-MAM101484 |  | Harvest | 10 |
| CNP-MAM101514 |  | Harvest | 14 |
| CNP-MAM101520 |  | Harvest | 16 |
| CNP-MAM101522 |  | Harvest | 9 |
| CNP-MAM101524 |  | Harvest | 6 |
| CNP-MAM101525 |  | Harvest | 11 |
| CNP-MAM101534 |  | Harvest | 6 |
| CNP-MAM101536 |  | Harvest | 9 |
| CNP-MAM101544 |  | Harvest | 5 |
| CNP-MAM101546 |  | Harvest | 6 |
| CNP-MAM101554 |  | Harvest | 5 |
| CNP-MAM101555 |  | Harvest | 5 |
| CNP-MAM101556 |  | Harvest | 6 |
| CNP-MAM101567 |  | Postharvest | 12 |
| CNP-MAM101577 |  | Harvest | 10 |
